# Automated planning architectures for online adaptive radiotherapy: A narrative review of the ethos and unity/Monaco platforms

**DOI:** 10.1002/acm2.70797

**Published:** 2026-09-14

**Authors:** Songye Cui, Maria F. Chan, Dandan Zheng

**Affiliations:** ^1^ Department of Medical Physics Memorial Sloan Kettering Cancer Center New York New York USA; ^2^ Department of Radiation Oncology, Wilmot Cancer Institute University of Rochester Medical Center Rochester New York USA

**Keywords:** adaptive radiotherapy, automated treatment planning, CBCT‐guided radiotherapy, hierarchical optimization, MR‐guided radiotherapy, online adaptive radiotherapy, treatment plan optimization

## Abstract

Online adaptive radiotherapy (OART) modifies treatment plans using anatomy‐of‐the‐day imaging, but successful clinical implementation depends on automated planning strategies that can generate high‐quality plans within the constraints of an online workflow. This narrative review compares the automated planning architectures of the Varian Ethos and Elekta Unity/Monaco systems using a targeted literature search through July 2026. Ethos generates a daily adapted plan through goal‐prioritized supervisory optimization governed by an unchanged clinical directive. Unity/Monaco provides Adapt‐to‐Position (ATP) and Adapt‐to‐Shape (ATS) pathways, with methods ranging from reference‐segment recalculation and refinement to full online replanning with newly optimized fluence and segments. Rather than representing opposing planning paradigms, these systems differ in how ordered clinical goals or reference‐plan information guide a selected adaptation method and in the degrees of freedom available within that method. Representative workflow and implementation studies are reviewed to provide clinical context, but they do not support direct comparisons of platform performance or superiority. MRIdian is acknowledged as another established MR‐guided adaptive platform, although its planning architecture is beyond the scope of this focused review. Accordingly, the comparison is best understood by asking what guides the selected adaptation method and which degrees of freedom that method permits, rather than as a binary distinction between flexible and reference‐based planning. This framework provides a conceptual basis for interpreting current commercial OART systems and may help guide the future development and evaluation of automated adaptive planning technologies.

## 1 INTRODUCTION

Adaptive radiotherapy (ART) enables modification of treatment delivery in response to changes occurring during the course of radiotherapy.^1^, ^2^, ^3^, ^4^, ^5^, ^6^ By incorporating updated imaging information, ART aims to reduce geometric uncertainty, maintain target coverage, and limit unnecessary irradiation of normal tissues.

Among adaptive paradigms, online adaptive radiation therapy (OART) has attracted particular interest because adaptation is performed immediately prior to treatment delivery using anatomy‐of‐the‐day imaging, as well as due to the growing availability of more streamlined, clinically integrated OART platforms.^4^, ^6^ Compared with offline adaptation, OART minimizes the time between imaging and irradiation, thereby reducing the impact of anatomical discrepancies.^4^, ^6^ By updating the treatment plan to match daily anatomy, OART is particularly valuable for patients with highly variable anatomy, where day‐to‐day adaptation can meaningfully improve alignment between planned and delivered dose.^4^, ^6^

Despite these theoretical advantages, widespread adoption of OART has historically been limited by workflow feasibility. Online adaptation requires image acquisition, contour generation or propagation, electron‐density assignment, treatment‐plan optimization, dose verification, and quality assurance while the patient remains immobilized on the couch. These workflows remain resource‐intensive and often require physician contour review, dosimetric plan adjustment, physicist quality assurance, and other human oversight, limiting scalability.^4^, ^6^, ^7^

Consequently, automation of treatment planning is important for the practical implementation and scalability of OART. Automated treatment planning has encompassed knowledge‐based planning (KBP),^8^, ^9^ multicriteria optimization (MCO),^10^, ^11^, ^12^ and more recent artificial intelligence (AI)‐driven methods such as deep learning for spatial dose prediction and reinforcement learning or generative models to emulate complex clinical decision‐making.^13^

Despite this broad body of work, focused analyses of automated planning as implemented in commercially deployed OART systems remain relatively sparse in the peer‐reviewed literature.^5^ Most prior studies^4^, ^6^, ^14^, ^15^ emphasize component‐level developments—such as optimization algorithms, knowledge‐based modeling, or AI‐based segmentation—rather than the integrated system architectures that govern adaptive planning behavior in clinical practice. This limitation reflects both the proprietary nature of commercial platforms and the complexity of tightly coupled imaging, optimization, and delivery workflows in online adaptation.

Recent discussions have also emphasized the need for more structured and collaborative frameworks to guide the development and clinical integration of automation in adaptive radiotherapy.^7^ While substantial progress has been made at the level of individual algorithms and components, the lack of a unified system‐level perspective continues to limit consistent interpretation and implementation of automated planning strategies across platforms.

This review is intended as a narrative review of commercial automated planning architectures in two representative online adaptive radiotherapy systems. Representative workflow and implementation evidence is included to provide clinical context for understanding how these planning architectures are deployed in practice, rather than to perform a comprehensive comparison of workflow efficiency or clinical implementation.

## 2 SCOPE AND LITERATURE SELECTION

A targeted PubMed and Europe PMC search was performed through July 12, 2026, supplemented by backward reference screening. Search concepts included online adaptive radiotherapy, Ethos, Unity, MR‐Linac, MRIdian, Monaco, automated planning, hierarchical optimization, segment aperture morphing, Adapt‐to‐Position, Adapt‐to‐Shape, workflow time, staffing, adaptation rate, failure mode, quality assurance, and clinical implementation. Peer‐reviewed technical descriptions and clinical cohorts reporting architecture or operational experience were prioritized. Vendor documents were used only when a proprietary function was not described adequately in peer‐reviewed sources. Because screening was targeted rather than exhaustive and no formal risk‐of‐bias assessment or meta‐analysis was performed, the evidence synthesis should be interpreted as narrative and hypothesis‐generating.

Statements are separated into three evidentiary categories: peer‐reviewed observations, disclosed vendor or technical descriptions, and interpretation of those descriptions within the proposed framework. Descriptions of proprietary commercial algorithms are restricted to publicly disclosed functionality. Assertions regarding flexibility, stability, convergence, robustness, computational efficiency, or scalability are treated as method‐specific interpretations unless supported by comparative evidence.

The focus on Ethos and Unity/Monaco also defines the review's boundaries. These systems were selected for detailed comparison because peer‐reviewed and technical descriptions disclose enough information about objective handling, optimization initialization, and segment generation to support analysis at a similar architectural level. MRIdian is another established commercial MR‐guided radiotherapy platform and, in its linac configuration, a distinct MR‐linac option; commercial OART also includes other photon workflows and emerging online adaptive proton approaches.^16^, ^17^, ^18^, ^19^ Peer‐reviewed MRIdian workflow evidence is included below to acknowledge that option and contextualize MR‐guided treatment times, but MRIdian is not used as a surrogate for Unity/Monaco and its optimizer architecture is not compared in equivalent depth. A full three‐platform technical review would require a separate analysis of MRIdian‐specific objective handling, optimization, delivery, and quality assurance. The proposed framework may assist comparison beyond the two emphasized systems, but it is not a comprehensive taxonomy of commercial OART.

## 3 CONCEPTUAL FRAMEWORK FOR AUTOMATED ADAPTIVE PLANNING

The comparison uses three interdependent questions: (1) how clinical intent is represented, (2) which degrees of freedom are available in the selected daily adaptation method, and (3) how imaging, contouring, dose calculation, delivery, staffing, and quality assurance constrain implementation.

### 3.1 Encoding clinical intent

Inverse treatment planning translates clinical objectives, such as target coverage and organ‐at‐risk (OAR) sparing, into mathematical optimization functions. Conventional planning often uses weighted‐sum formulations in which competing objectives are balanced through numerical weight adjustment.^20^ Such formulations are flexible, but clinical priorities may be implicit in the selected weights and may require iterative tuning.

Automated adaptive systems differ in how they represent clinical intent and initialize daily optimization. Ethos uses ordered clinical goals with priority‐sensitive plan‐quality evaluation and supervisory objective adjustment.^21^, ^22^ In Unity/Monaco, Adapt‐to‐Position (ATP) warm starts use reference‐plan dose and segments, whereas Adapt‐to‐Shape (ATS) reoptimization uses pre‐treatment planning objectives.^23^, ^24^ Intent representation and adaptation depth should therefore not be conflated: the information guiding Unity optimization is itself method‐dependent, while the selected method separately determines whether reference segments are retained or new fluence and segments are generated; Ethos applies an ordered clinical directive to daily plan generation without reference‐segment initialization.

### 3.2 Adaptation as a spectrum rather than a binary

Daily adaptation spans a spectrum of available degrees of freedom. One end regenerates the optimization problem from current anatomy under a fixed clinical policy, as in the Ethos adapted‐plan workflow.^21^, ^22^ Reference‐based methods reuse or morph approved apertures and then recalculate or refine segment weights or shapes. Unity includes these methods, particularly in ATP, but its ATS workflow also permits methods that discard the reference segments, optimize new fluence, generate new segments, and perform full online replanning.^23^ Therefore, Ethos and Unity should not be classified as mutually exclusive global versus reference‐anchored paradigms. A more accurate comparison asks which adaptation method is invoked, how its degrees of freedom are initialized, and how clinical objectives are enforced.

### 3.3 Workflow and physical modeling constraints

Adaptive optimization is embedded within imaging, contouring, dose calculation, quality assurance, and delivery constraints.^4^, ^5^ MR guidance provides high soft‐tissue contrast and intrafraction visualization but requires magnetic‐field‐aware dose calculation and longer imaging or monitoring tasks in many workflows.^25^ CBCT workflows may require more contour correction when soft‐tissue contrast or artifacts limit segmentation.^21^, ^22^, ^26^, ^27^, ^28^ Delivery also constrains available planning methods: fixed‐field or arc delivery, step‐and‐shoot segmentation, monitor‐unit limits, and field size affect optimization and verification. These factors create a time‐constrained clinical process, but they do not identify a universal rate‐limiting step.

**FIGURE 1:**
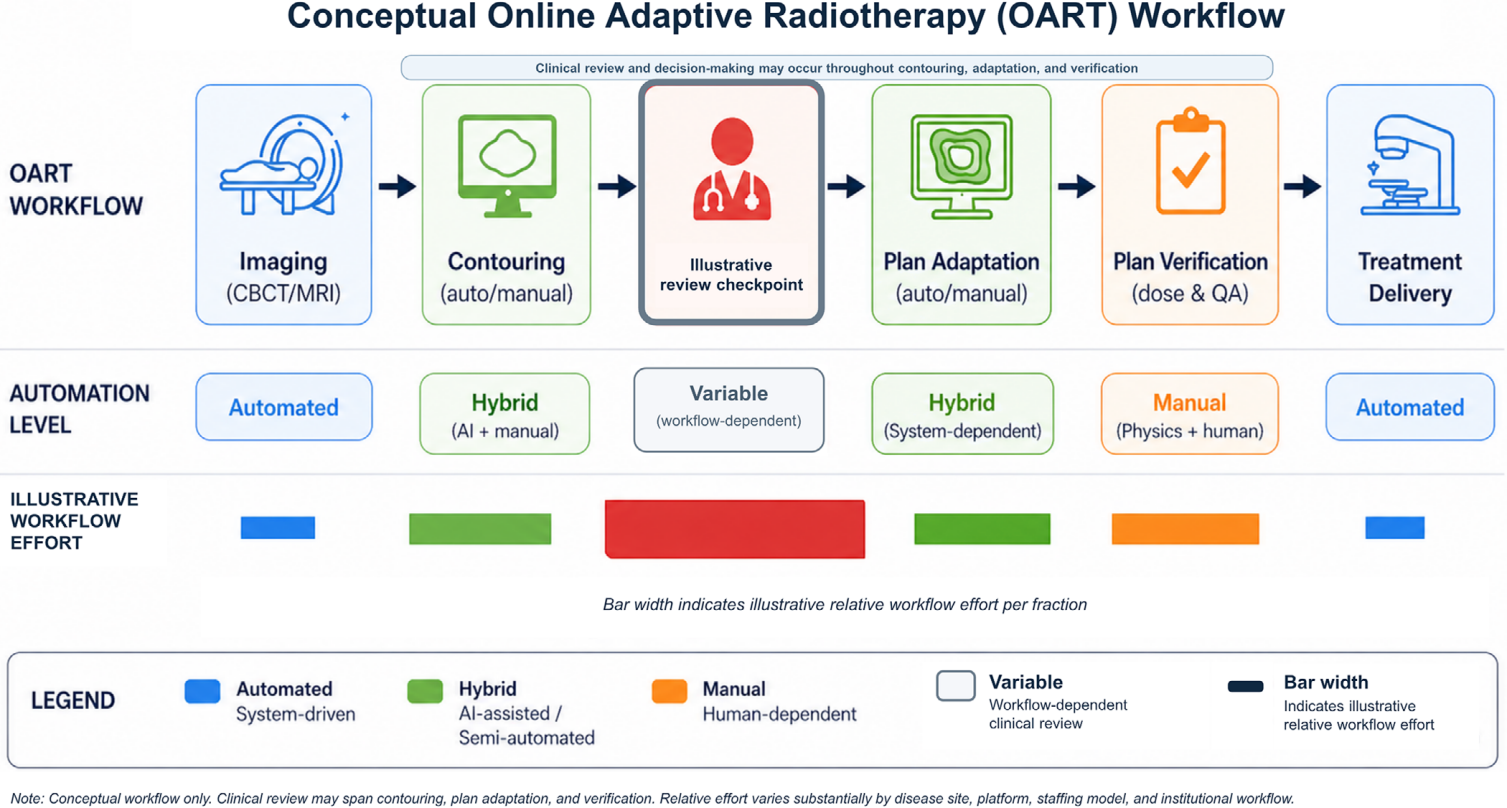
Conceptual OART workflow. The review panel is an illustrative checkpoint; clinical review and decision‐making may span contouring, plan adaptation, and plan verification. Automation level and the relative contribution of each component vary substantially among disease sites, platforms, staffing models, and institutional workflows; the illustrated workflow effort is qualitative and does not identify a universal bottleneck.^16^, ^17^, ^26^, ^27^, ^28^, ^29^, ^30^, ^31^, ^32^, ^33^

Figure 1 presents a conceptual OART workflow comprising imaging, contouring, plan adaptation, plan verification, and treatment delivery, with the review panel shown only as an illustrative checkpoint. Clinical review and decision‐making may span contouring, adaptation, and verification rather than forming a single fixed stage. Automation level and the relative effort associated with each component are workflow dependent and may vary substantially by disease site, platform, staffing model, and institutional implementation; the illustrated effort is not a universal timing estimate or bottleneck assignment.^16^, ^17^, ^26^, ^27^, ^28^, ^29^, ^30^, ^31^, ^32^, ^33^ Physician review can be important in some implementations, but trained radiation therapist‐led workflows and optimized contour‐generation strategies can reduce daily physician intervention in selected indications.^28^, ^32^

### 3.4 Representative workflow evidence as clinical context

Operational performance cannot be inferred from optimization architecture alone. Table 1 therefore presents a deliberately limited set of representative implementation cohorts rather than a comparative workflow review. Reported endpoints are not interchangeable: some studies measure contouring and replanning, whereas others measure total treatment or adaptive‐session time. Standardized throughput is not compared because operating hours, adaptive‐fraction mix, parallel work, and staffing denominators were reported inconsistently.

**TABLE 1:** Representative clinical workflow and implementation evidence included for context.

Study/Platform/Cohort	Timing Reported	Operational or Clinical Findings	Critical Appraisals
Byrne et al.^26^Ethos; prostate 184 clinical and 182 simulated fractions	Mean 19 min for online recontouring and replanning in the simulated workflow; not total clinical room time.	Adapted plan selected in 95% of analyzed fractions; more listed goals met in 78% of analyzed fractions.	Single‐center early experience with mixed clinical and simulated analyses; timing definition limits cross‐study comparison. The institution was a Varian Adaptive Intelligence Consortium member and equipment partner; three authors reported Varian honoraria.
Zheng et al.^27^Ethos; pelvis More than 1000 sessions; 50 patients	Timing was not the primary endpoint.	The authors reported > 99% of sessions completed successfully and 9 aborted. System‐, patient‐, and execution‐related failure modes required mitigation and fallback planning.	Retrospective single‐institution failure analysis; detailed event totals do not fully reconcile with the narrative summary, so counts are presented as author reported. One author had an open online adaptive radiotherapy trial under a Varian research agreement; Varian provided in‐kind Ethos emulator access.
Kalinauskaite et al.^28^ Ethos; prostate 613 fractions; 34 patients	Median 24 min in‐room time.	A five‐influencer contour workflow reduced target corrections to 11% versus 51‐61% with other workflows, supporting reduced physician involvement in this setting.	Implementation‐specific workflow comparison without a cross‐platform comparator. Departmental financial, technical, and educational support from Varian was disclosed.
Westerhoff et al.^32^ Unity 4942 fractions; 645 patients	Mean total: prostate ATP 15 min; prostate ATS 39 min; ATS 34‐41 min across other reported sites.	Learning reduced online adaptation by 4‐8 min in several ATS groups; an RTT‐led model did not significantly increase total treatment time.	Single institution with heterogeneous sites and methods; no Ethos comparator. The cohort was analyzed within MOMENTUM, an academic‐industry registry financially supported by Elekta.
Güngör et al.^16^ MRIdian 774 adaptive / 962 total fractions	Median net total treatment time 45 min.	Session components varied by anatomical site.	MRIdian, not Unity/Monaco; included only as contextual MR‐guided workflow evidence.

*Note*: This table presents selected representative studies as clinical context, not a comprehensive workflow comparison. Timing definitions are those used by the cited authors and are not interchangeable. Standardized patient‐throughput comparisons were not available. Manufacturer relationships or support reported by the cited studies are summarized in the critical‐appraisal column. The MRIdian row provides contextual MR‐guided workflow evidence and must not be attributed to Unity/Monaco architecture.

Abbreviations: ATP, Adapt‐to‐Position; ATS, Adapt‐to‐Shape; RTT, radiation therapist. MR, magnetic resonance.

The selected studies provide clinical context for why automated‐planning architecture matters. For Ethos, Byrne et al. reported 95% adapted‐plan selection across simulated and clinical fractions and a mean 19 min for online recontouring and replanning in the simulated workflow; Zheng et al. reported in the article narrative that > 99% of sessions were successfully delivered and nine were aborted; and Kalinauskaite et al. reported that a modified contour workflow reduced target corrections to 11% from 51–61% in that setting.^26^, ^27^, ^28^ For MR‐guided radiotherapy, Westerhoff et al. found method‐ and site‐dependent timing, including mean total times of 15 min for prostate ATP and 39 min for prostate ATS.^32^ Güngör et al. reported a median net total treatment time of 45 min for MRIdian, included only to illustrate broader MR‐guided workflow variability rather than to characterize Unity/Monaco.^16^ The Zheng summary values are presented as author reported because detailed event totals within that article are not fully internally consistent. These observations are implementation‐specific, and manufacturer relationships or support reported by the cited studies are summarized in Table 1; none supports platform ranking.^27^

## 4 INTENT‐CONSISTENT SUPERVISORY RE‐OPTIMIZATION: THE ETHOS PLATFORM

### 4.1 Workflow architecture

The Ethos system implements CBCT‐based OART within a strictly time‐constrained on‐couch workflow.^21^, ^22^ Daily adaptation begins with CBCT acquisition to capture the anatomy‐of‐the‐day. Artificial intelligence–based segmentation identifies selected “influencer” organs directly on the CBCT.^21^ These structures guide deformable propagation of target volumes from the planning CT, producing a session‐specific patient model.^21^ Clinician review and limited contour correction are performed to ensure geometric accuracy within a clinically acceptable time window.^21^

Two plans are automatically generated^21^:

- A scheduled plan, representing recalculated dose of the reference plan on daily anatomy
- An adapted plan, produced through de novo supervisory optimization using the unchanged hierarchical clinical goal template

For standard‐CBCT sessions, dose is calculated on a synthetic CT obtained by deforming planning‐image voxel values to the session anatomy. For HyperSight™ CBCT sessions, session‐image pixels are used directly within the session‐image field of view, while planning‐image voxel information is propagated by rigid registration outside that field of view.^22^ After clinical review, the scheduled and adapted plans are compared and the final plan is selected for treatment.^21^, ^22^

This workflow is designed to automate segmentation support and plan generation while preserving a consistent clinical directive across fractions. Representative cohorts nevertheless show that contour correction, plan selection, and fallback needs remain implementation‐dependent.^26^, ^27^, ^28^

### 4.2 Intelligent optimization engine (IOE): Hierarchical goal translation and supervisory control

The Intelligent Optimization Engine (IOE) functions as an orchestration layer above the underlying inverse planning solver.^21^, ^22^ Related automated re‐optimization approaches^34^, ^35^, ^36^ have similarly used feedback from achieved dose metrics or DVH objectives to guide objective adjustment during replanning. Clinical goals defined in the RT Intent module are supplied as an ordered priority list and translated into optimization objectives.^21^, ^22^, ^37^ The 2020 public description maps achieved goal metrics to goal‐specific quality functions and *P*‐values and describes increasing the cost contribution associated with the lowest‐performing *P*‐value.^21^ The 2025 algorithms guide instead describes an overall plan‐quality value, P, obtained by summing continuous, priority‐sensitive quality values across the individual clinical goals; IOE monitors intermediate plans and modifies photon‐optimization objectives expected to improve overall *P*.^22^ These are software‐generation‐specific disclosures and should not be presented as an identical mechanism. In both descriptions, the ordered goals remain active within a global optimization and guide supervisory adjustment rather than being solved as strict sequential lexicographic subproblems.^21^, ^22^

### 4.3 Structural preprocessing and conflict resolution

Prior to optimization, IOE performs automated structure preprocessing to address geometric conflicts.^21^, ^22^ When overlaps between targets or between targets and OARs create incompatible goals, optimization structures are modified according to priority order. Cropping operations and margin adjustments are applied to enforce physically realizable dose gradients in regions of overlap while preserving hierarchical intent. Thus, hierarchy influences not only optimization weighting but also the geometric definition of optimization volumes. This preprocessing step reduces infeasible trade‐offs before global optimization begins.

### 4.4 Auxiliary objectives and plan quality controls

Beyond user‐defined clinical goals, IOE automatically introduces auxiliary objectives or goals to maintain overall plan quality.^21^, ^22^ These may include normal‐tissue control structures, global maximum‐dose controls, auxiliary target or organ goals, and controls on plan complexity. The treatment of RapidPlan guidance is version‐specific. The 2020 IOE description adds predicted DVH information as low‐weight line objectives without an explicit clinical‐goal priority.^21^ In the 2025 IOE description, the lower DVH‐estimate band is converted into an additional clinical goal and placed in priority group 2 below user‐supplied goals.^22^ In both descriptions, user‐defined clinical goals remain the principal drivers of optimization.

### 4.5 Conceptual characterization

Based on disclosed descriptions, Ethos can be characterized as goal‐prioritized supervisory global optimization. Ordered clinical goals guide objective construction and modification during daily optimization that is not initialized from reference‐plan segment topology.^21^, ^22^ The disclosed plan‐quality and RapidPlan mechanisms vary between the 2020 and 2025 descriptions. This distinguishes Ethos adapted‐plan generation from Unity methods that retain reference segments, but not from Unity ATS methods that generate new fluence and segments. Whether the Ethos architecture produces better plan quality, robustness, flexibility, or efficiency than a specific Unity method has not been established by direct comparative evidence.

**FIGURE 2:**
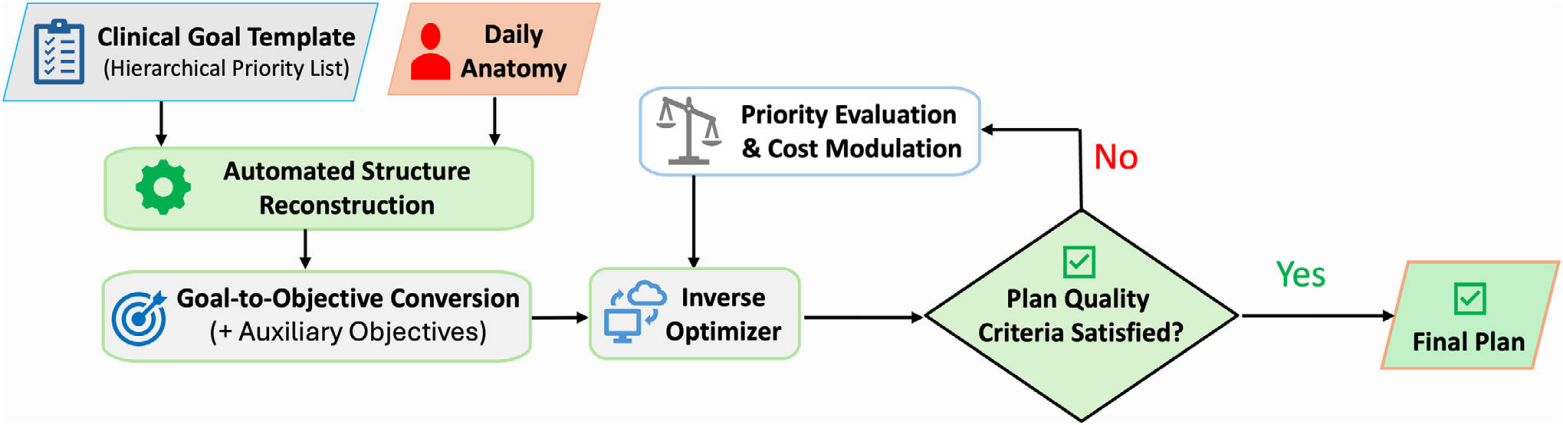
High‐level supervisory optimization in the Ethos adaptive planning workflow. Specific plan‐quality and objective‐modification mechanisms vary by software generation.^21^, ^22^

Figure 2 summarizes the shared high‐level supervisory architecture disclosed across versions. An ordered clinical‐goal template and daily anatomy are translated into optimization structures and objectives; IOE evaluates intermediate plan quality and modifies optimizer objectives until stopping criteria are met.^21^, ^22^ The figure does not imply that the 2020 goal‐level *P*‐value rule and the 2025 overall plan‐quality formulation are identical.

## 5 WORKFLOW‐CONDITIONAL ADAPTIVE PLANNING: THE UNITY/MONACO PLATFORM

### 5.1 Workflow architecture

The Elekta Unity MR‐Linac integrates a 1.5 T MRI scanner with a linear accelerator to enable MR‐guided OART.^23^ Although MR imaging provides superior soft‐tissue contrast and enables intra‐fraction monitoring, the adaptive workflow remains subject to strict temporal constraints: imaging, contour propagation and correction, plan adaptation, dose verification, and delivery must be completed within a single on‐couch session.

Daily adaptation begins with volumetric MR acquisition. Two principal adaptive workflows are available:

- Adapt‐to‐Position (ATP): rigid registration is applied to align the pre‐treatment data with the daily anatomy, and plan adaptation is performed without modification of contours.^23^, ^24^
- Adapt‐to‐Shape (ATS): deformable registration is used to propagate contours to the daily MRI, followed by manual review and correction prior to plan adaptation.^23^, ^24^

Unity provides six disclosed plan adaptation methods that can be used within ATP or ATS.^23^ Methods are defined as follows: A, original segments; B, adapted segments using segment aperture morphing (SAM); C, weight optimization from reference segments; D, weight optimization from newly optimized fluence and segments; E, weight‐and‐shape optimization from reference segments; and F, weight‐and‐shape optimization from newly optimized fluence and segments (full online replanning).^23^ ATP permits A, B, C, and E, whereas ATS permits all six methods. Thus, optimization depth is a workflow and method choice rather than a single platform‐wide property.

### 5.2 ATP: Reference‐data and reference‐segment adaptation

ATP rigidly registers the pre‐treatment CT to the daily MRI, updates the virtual isocenter on the reference data, and adapts the plan without daily contour editing.^23^ Available methods may recalculate original segments, morph apertures using SAM, optimize segment weights, or optimize both weights and shapes. The plan is optimized and evaluated on the reference CT and contours rather than on edited anatomy‐of‐the‐day contours.^23^ For warm‐start weight or weight‐and‐shape optimization, the objective function seeks to reproduce or improve the reference‐plan dose distribution while retaining the selected reference‐segment representation.^24^

These reference‐based methods can be faster because they avoid online contour adaptation and fluence reoptimization. Their adequacy is case‐specific. In the five illustrative cases reported by Winkel et al., faster ATP methods did not consistently satisfy constraints when recalculated on daily anatomy, whereas performance varied with anatomy and method.^23^ This evidence supports method selection and verification, not a general conclusion that reference‐based adaptation is intrinsically stable or sufficient.

### 5.3 ATS: Anatomy‐of‐day optimization through full replanning

ATS propagates contours deformably to the daily MRI, permits online review and correction, applies structure‐based electron‐density assignments for calculation on the daily dataset, and performs reoptimization using the pre‐treatment planning objectives.^23^ ATS may retain reference segments for recalculation or weight/shape refinement, but it may also optimize new fluence and generate a new segment set. In the disclosed method taxonomy, method F performs fluence reoptimization followed by segmentation and segment weight‐and‐shape optimization and is equivalent to full online replanning.^23^ Warm‐start or SAM descriptions therefore apply to selected methods, not to the entire Unity/Monaco adaptive architecture.

**FIGURE 3:**
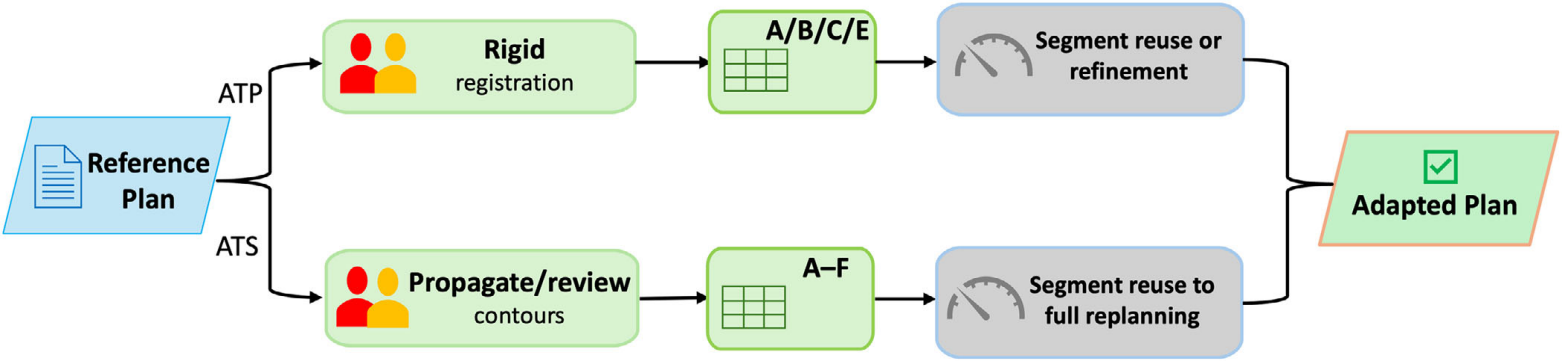
Unity/Monaco adaptation spectrum. (a) original segments; (b) SAM‐adapted segments; (c) weight optimization from reference segments; (d) weight optimization from newly optimized fluence and segments; (e) weight‐and‐shape optimization from reference segments; and (f) weight‐and‐shape optimization from newly optimized fluence and segments (full online replanning). ATP permits a–c, e, whereas ATS permits a–f.^23^

Figure 3 shows this workflow‐dependent spectrum. ATP uses rigid registration and reference‐data methods. ATS adds anatomy‐of‐day contour propagation and review, then permits either segment‐based adaptation or methods extending to full online replanning. Final calculation and approval remain coupled to the selected method and local quality‐assurance workflow.

### 5.4 Dose modeling and physical considerations

MR‐guided adaptive workflows introduce unique physical‐modeling requirements. Hissoiny et al. introduced GPUMCD as a GPU‐oriented Monte Carlo dose‐calculation platform.^38^

In the Unity workflow, the transverse magnetic field alters secondary‐electron trajectories and dose deposition, particularly at air‐tissue interfaces.^39^ Chuter et al. describe GPUMCD accounting for the 1.5 T field in second‐stage segment‐shape optimization and final dose calculation.^39^ In first‐stage fluence optimization, Monaco can use either a faster pencil‐beam engine that does not model the magnetic field or GPUMCD.^39^ ATP calculates dose on the rigidly registered pre‐treatment CT, whereas ATS calculates on the daily MRI using structure‐based electron‐density assignments; in the Unity concept description, each structure is assigned the average electron density of the corresponding pre‐treatment CT contour.^23^ These modeling choices affect optimization time, but their independent effect on total session duration has not been established.^39^

### 5.5 Delivery coupling and practical constraints

Unity delivers step‐and‐shoot IMRT. This delivery design is compatible with segment reuse and morphing, but it also supports ATS methods that create new fluence and segments.^23^ The delivery technique therefore shapes the available plan representation without requiring every adaptive method to preserve reference segment topology.

When a reference‐segment method is selected, substantial anatomical change may exceed the useful degrees of freedom and motivate a more extensive ATS method or offline replanning. ATS full replanning expands the available optimization freedom but adds contour‐review and optimization tasks. The clinical trade‐off is therefore method‐, site‐, and workflow‐specific rather than a fixed platform‐level balance between flexibility and stability.

Unity‐specific implementation data reinforce that workflow choice matters. In 4942 fractions from 645 patients, Westerhoff et al. reported mean total treatment times of 15 min for prostate ATP and 39 min for prostate ATS, with additional method‐ and site‐dependent variation.^32^ These single‐institution values should not be treated as universal ATP or ATS durations.

Unity/Monaco is therefore better characterized as a workflow‐conditional adaptation system than as a uniformly reference‐anchored optimizer. Its practical performance depends on ATP versus ATS selection, the chosen optimization method, contour‐review burden, disease site, staff roles, verification imaging, and local acceptance criteria.

## 6 COMPARATIVE ANALYSIS OF ADAPTIVE PLANNING ARCHITECTURES

### 6.1 Clinical intent encoding and trade‐off enforcement

Ethos and Unity/Monaco differ in both supervisory policy and method‐dependent initialization. Ethos applies an ordered clinical directive and plan‐quality evaluation to daily adapted‐plan generation.^21^, ^22^ Unity ATP warm‐start methods seek to reproduce or improve the reference‐plan dose distribution from reference segments, whereas ATS reoptimization uses the pre‐treatment planning objectives.^23^, ^24^ The comparison is therefore between different intent or reference representations and method‐specific adaptation choices, not a single hierarchical‐versus‐weighted platform binary.

### 6.2 Optimization initialization and available degrees of freedom

The Ethos adapted plan is regenerated from the clinical directive and daily anatomy without inheriting reference‐plan segment topology.^21^, ^22^ In Unity, the available degrees of freedom depend on the selected method: methods A, B, C, and E retain or transform reference segments, whereas ATS methods D and F optimize new fluence and generate new segments; method F is full online replanning.^23^ Architecture‐derived claims about search‐space breadth or convergence must therefore be made at the method level. No standardized head‐to‐head study demonstrates that one platform is more flexible, stable, robust, or efficient.

### 6.3 Automation depth and workflow integration

Automation occurs at multiple layers: image processing, contour generation, objective construction, optimization control, plan evaluation, and verification. Ethos integrates AI‐supported influencer segmentation with supervisory plan generation, but representative cohorts still report contour edits, plan selection, and fallback needs.^26^, ^27^, ^28^ Unity automates registration and plan adaptation while the required online contour review depends on ATP versus ATS; radiation therapist‐led models have been implemented without a statistically significant increase in total treatment time in selected Unity workflows.^32^ Automation depth therefore cannot be inferred from the optimizer alone.

### 6.4 Physical modeling and delivery coupling

Both systems support clinically feasible online adaptation, but scalability is an observed implementation outcome rather than an optimizer property. Imaging quality, contour policy, delivery technique, dose calculation, plan verification, staff credentialing, patient tolerance, and fallback procedures all affect throughput and safety.^16^, ^17^, ^26^, ^27^, ^28^, ^29^, ^30^, ^31^, ^32^, ^33^

### 6.5 Conceptual synthesis

A two‐axis conceptual map uses independent dimensions: the information that guides optimization (ordered clinical goals, pre‐treatment objectives, or reference‐plan dose/segments) and adaptation freedom (reference‐segment reuse through full replanning). Ethos occupies a policy‐driven daily reoptimization pathway governed by ordered clinical goals. Unity's position is method‐dependent on both axes: ATP warm‐start methods use reference‐plan dose and segment information, whereas ATS reoptimization uses pre‐treatment objectives and may extend to new fluence and segments. Table 2 summarizes disclosed differences without assigning platform superiority.

**TABLE 2:** Comparison of disclosed automated planning architectures and adaptation options in OART.

Features	Varian ethos	Elekta unity/Monaco
Adaptation model	CBCT‐guided daily adapted‐plan generation under an unchanged hierarchical clinical directive.	MR‐guided ATP/ATS workflows with method‐dependent adaptation depth.
Clinical intent encoding	Ordered goals guide supervisory optimization. The 2020 disclosure uses goal‐level quality functions and *P*‐values; the 2025 guide uses an overall priority‐sensitive plan‐quality function.^21^, ^22^	Method dependent: ATP warm‐start methods use the reference‐plan dose distribution and reference segments; ATS reoptimization uses the pre‐treatment planning objectives.^23^, ^24^
Primary imaging	CBCT	1.5 T MRI
Daily anatomy and contours	AI influencer segmentation plus deformable target propagation, followed by online review and editing.^21^	ATP: rigid registration without daily contour editing. ATS: deformable propagation with online review and editing.^23^, ^24^
Adaptation options	Scheduled‐plan recalculation and a de novo adapted plan generated from the directive and daily anatomy.	ATP uses reference‐data methods A/B/C/E. ATS permits A‐F, including new‐fluence methods D/F; method F is full online replanning.^23^
Reference‐segment dependence	The adapted plan is not initialized from reference‐plan segment topology.^21^, ^22^	Method dependent: segments are retained or transformed in A/B/C/E and discarded and regenerated in D/F^23^
Delivery	Fixed‐field IMRT or VMAT	Step‐and‐shoot IMRT
Final dose calculation	Acuros XB^22^	GPUMCD, configured to account for the 1.5 T magnetic field.^38^, ^39^
Workflow‐sensitive factors	Directive robustness, image and contour quality, plan review, verification/QA, staffing, and fallback policy.	ATP/ATS and method selection, ATS contour review, dose calculation/QA, staffing, monitoring, and fallback policy.
Evidence boundary	No standardized direct architecture or outcome comparison establishes platform superiority; observed performance is site‐ and implementation‐dependent.	No standardized direct architecture or outcome comparison establishes platform superiority; observed performance is site‐ and implementation‐dependent.

*Note*: MRIdian is a distinct MR‐guided option and is not represented in this two‐platform architecture table; selected MRIdian workflow evidence is summarized in Table 1. Methods: A, original segments; B, SAM‐adapted segments; C, weight optimization from reference segments; D, weight optimization from newly optimized fluence and segments; E, weight‐and‐shape optimization from reference segments; and F, weight‐and‐shape optimization from newly optimized fluence and segments (full online replanning).

Abbreviations: ATP, Adapt‐to‐Position; ATS, Adapt‐to‐Shape; CBCT, cone‐beam computed tomography; GPUMCD, GPU‐oriented Monte Carlo dose‐calculation platform; IMRT, intensity‐modulated radiation therapy; MR, magnetic resonance; MRI, magnetic resonance imaging; OART, online adaptive radiotherapy; QA, quality assurance. AI, artificial intelligence; SAM, segment aperture morphing; T, tesla; VMAT, volumetric modulated arc therapy.

### 6.6 Evidence limitations and interpretive boundaries

The evidence base has four major limitations. First, commercial algorithms are incompletely disclosed, and vendor documentation remains necessary for some implementation details. Second, clinical reports are predominantly single‐platform and single‐institution cohorts with heterogeneous disease sites and endpoints. Third, workflow, imaging, and learning effects limit the transferability of reported timing or staffing observations.^16^, ^26^, ^27^, ^28^, ^32^ Fourth, adapted plans often improve target coverage or goal attainment, but organ‐at‐risk gains are inconsistent and clinical‐outcome evidence is limited.^26^, ^29^, ^31^ Consequently, the literature supports clinical feasibility and method‐specific observations, but not a universal platform ranking.

## 7 EMERGING DIRECTIONS IN AUTOMATED ADAPTIVE PLANNING

The concepts in this section are hypotheses and research directions rather than established clinical conclusions. They are included to identify questions raised by current systems: how intent should be represented, when adaptation depth should change, how uncertainty should be incorporated, and where human review adds value.

### 7.1 Supervisory and hierarchical clinical intent encoding

A fundamental requirement of automated adaptive planning is the explicit representation of clinical intent. Conventional inverse planning expresses competing objectives through weighted‐sum formulations, requiring manual adjustment of relative importance to achieve acceptable trade‐offs.^20^ While effective, such approaches embed clinical priorities implicitly within numerical weights and may require iterative tuning.

Feedback‐driven supervisory optimization provides one way to make priority handling more explicit.^21^, ^34^, ^35^ These systems use achieved dose metrics or DVH objectives to guide predefined objective adjustment, although their exact control logic varies. Such approaches may improve interpretability of the encoded policy, but reproducibility and clinical benefit still depend on template design, anatomy, and validation.

PortPy provides an open‐source planning‐and‐optimization environment and benchmark data.^40^ Expedited Constrained Hierarchical Optimization (ECHO) formalizes planning goals through a prioritized hierarchy and combines constrained optimization with plan evaluation.^41^, ^42^ Although PortPy can support evaluation of ECHO, it is not the primary source for that architecture. ECHO is conceptually relevant to OART because online planning requires consistent and time‐bounded decisions; however, these sources do not establish it as a commercial online‐adaptive workflow. Application to OART should therefore be treated as a testable extension rather than a demonstrated conclusion.

Future work could evaluate whether supervisory intent models can be combined with method selection, uncertainty checks, or bounded trade‐off exploration without reducing transparency or increasing on‐couch time.

A fixed hierarchy may also be suboptimal when anatomy‐of‐the‐day changes the relevance of competing OAR goals. For example, an OAR that drove the original priority order may move away from the high‐dose region while another becomes limiting. Dynamic prioritization could address this situation, but it would require prospective rules, auditability, and safeguards against inconsistent daily intent. This remains a hypothesis for evaluation.

### 7.2 Multi‐criteria optimization and trade‐off navigation

Multi‐criteria optimization (MCO) represents competing objectives through a Pareto surface rather than a single weighted objective.^10^, ^11^, ^12^ GPU‐based work in moderate‐scale problems suggests that plan‐pool generation can be accelerated,^43^ but this does not establish real‐time clinical feasibility across OART disease sites or platforms.

For OART, hypothesized uses include visualizing anatomy‐of‐day trade‐offs and allowing bounded navigation after automated baseline planning. These functions could make the cost of changing one objective more explicit, but their effects on session time, decision consistency, and safety require clinical evaluation.

A future hybrid system might therefore combine automated baseline generation with constrained Pareto navigation inside predefined limits. This is a proposed design direction, not evidence that such a workflow improves outcomes or efficiency.

### 7.3 Data‐driven and learning‐based planning paradigms

Knowledge‐based planning and machine‐learning models can predict dose distributions or objective parameters from prior data.^6^, ^8^, ^9^, ^44^, ^45^ In OART, proposed roles include initialization, achievable‐dose estimation, and identification of anatomy‐driven trade‐off changes. These models should be evaluated as decision support within a validated optimization and quality‐assurance framework rather than assumed to replace deterministic planning.

Domal et al. retrospectively reoptimized 23 adaptive fractions from 15 head‐and‐neck patients using deep‐learning‐predicted planning goals; 19 of 23 reoptimized plans were preferred in blinded physician review.^46^ The study also illustrated one prospective case, but it did not demonstrate routine live deployment. These preliminary single‐institution findings support further evaluation of predictive priors but do not establish general performance across institutions, disease sites, or commercial platforms.

Learning‐based methods also introduce distribution shift, institutional dependence, and interpretability concerns. A practical research question is whether deterministic constraints and prospective monitoring can bound these risks while preserving any efficiency gained from model‐based initialization or tuning.

### 7.4 Synthesis and outlook

These directions are complementary possibilities rather than a demonstrated transition path. Hierarchical intent models, Pareto representations, and data‐driven guidance could be combined, but each adds validation, governance, and workflow requirements that must be tested in clinical implementation.

Future systems should be evaluated on transparent intent representation, time‐bounded and auditable adaptation, reproducibility, failure recovery, and staff interaction. Hardware and imaging remain important, but software architecture should be compared through measured behavior rather than inferred advantage.

## 8 CONCLUSION

This focused narrative review compared automated adaptive planning through method‐dependent intent or reference representation, adaptation freedom, and workflow constraints. Ethos uses ordered clinical goals and supervisory plan‐quality evaluation to generate a daily adapted plan, with disclosed implementation details that vary by software generation. Unity/Monaco provides ATP and ATS workflows ranging from reference‐segment recalculation and warm‐start refinement to ATS full online replanning; ATP warm‐start methods use reference‐plan dose and segment information, whereas ATS reoptimization uses pre‐treatment planning objectives. Unity should therefore not be characterized uniformly as reference‐anchored or geometry‐constrained, and the comparison should not be reduced to a single hierarchical‐versus‐weighted platform binary.

Published cohorts demonstrate feasibility but not head‐to‐head platform superiority. Session time, staffing, adaptation frequency, contour burden, and failure modes vary with disease site and local implementation; target‐coverage gains are reported more consistently than OAR or clinical‐outcome gains. Future studies should use standardized timing definitions, report staffing and fallback events, specify the software generation and exact adaptation method, and separate vendor‐described function from independently validated performance. MRIdian remains an important distinct MR‐guided option, but its workflow results should not be attributed to Unity/Monaco architecture. Proposed hierarchical, multicriteria, and learning‐based extensions remain hypotheses until tested in such frameworks.

## AUTHOR CONTRIBUTIONS

**Songye Cui**: conceptualization, literature review, and original drafting. **Maria F. Chan and Dandan Zheng**: critical review and editing. All authors approved the final manuscript.

## FUNDING INFORMATION

This research was supported by the National Cancer Institute Cancer Center Support Grant P30 CA008748.

## CONFLICT OF INTEREST STATEMENT

The authors declare no conflicts of interest.

## ETHICS STATEMENT

Not applicable. This narrative review is based on previously published literature and does not report new studies involving human participants or animals.

## Data Availability

Data sharing is not applicable to this article because no new datasets were generated or analyzed during the current study; all sources are cited in the manuscript.
